# Perimenopausal symptoms in women with and without ADHD: A population-based cohort study

**DOI:** 10.1192/j.eurpsy.2025.10101

**Published:** 2025-09-04

**Authors:** Unnur Jakobsdóttir Smári, Unnur Anna Valdimarsdottir, Dora Wynchank, Maxime de Jong, Thor Aspelund, Arna Hauksdottir, Edda Bjork Thordardottir, Gunnar Tomasson, Johanna Jakobsdottir, Donghao Lu, Alicia Nevriana, Henrik Larsson, Sandra Kooij, Helga Zoega

**Affiliations:** 1 Centre of Public Health Sciences, Faculty of Medicine, University of Iceland, Reykjavik, Iceland; 2 Unit of Integrative Epidemiology, Institute of Environmental Medicine, Karolinska Institutet, Stockholm, Sweden; 3 Department of Epidemiology, Harvard T.H. Chan School of Public Health, Boston, MA, USA; 4 PsyQ, Expertise Centre Adult ADHD, The Hague, The Netherlands; 5 Department of Medical Epidemiology and Biostatistics, Karolinska Institutet, Stockholm, Sweden; 6 School of Medical Sciences, Örebro University, Örebro, Sweden; 7 Amsterdam University Medical Center, Department of Psychiatry, Amsterdam, The Netherlands

**Keywords:** ADHD, attention-deficit/hyperactivity disorder, perimenopausal symptoms, comorbidities, women

## Abstract

**Background:**

Knowledge of the impact of perimenopause on women with attention-deficit/hyperactivity disorder (ADHD) is lacking. We compared levels of perimenopausal symptoms and prevalence of severe perimenopausal symptoms among women with and without ADHD across age groups.

**Methods:**

In this cohort study, we used data from the population-based Stress-and-Gene-Analysis cohort study. ADHD diagnosis was self-reported at baseline and 5-year follow-up. At follow-up, we assessed ADHD symptoms using the Adult ADHD Self-Report Scale, perimenopausal symptoms (psychological, somatic, and urogenital) using Menopause Rating Scale (MRS), and general physical symptoms using Patient Health Questionnaire. We described mean scores and mean difference on MRS among women with and without ADHD with linear regression models and contrasted the prevalence of severe perimenopausal symptoms among women with and without ADHD, calculating prevalence ratios (PRs) with 95% confidence intervals (CIs) using modified Poisson regression models.

**Results:**

Women with ADHD (*n* = 535) had higher total perimenopausal symptom scores (18.0 vs. 13.0, *p* < 0.01) than women without ADHD (*n* = 4,857). The difference was most pronounced among women aged 35–39 years (19.0 vs. 12.5, *p* < 0.01). The prevalence of severe perimenopausal symptoms was significantly higher among women with ADHD compared to those without, both overall (54.2% vs. 30.1%, PR = 1.80, 95% CI = 1.64–1.98) and on all subdimensions (psychological: 58.6% vs. 36.0%, PR = 1.63, 95% CI = 1.51–1.76; somatic: 30.4% vs. 13.9%, PR = 2.20, 95% CI = 1.88–2.57; uro-genital: 43.2% vs. 27.5%, PR = 1.57, 95% CI = 1.40–1.77).

**Conclusion:**

Women with ADHD have higher prevalence of severe perimenopausal symptoms. These symptoms present at an earlier age than among women without ADHD, indicating an earlier onset age of perimenopause in ADHD.

## Introduction

Women remain underrepresented in research on attention-deficit/hyperactivity disorder (ADHD), and knowledge of the impact of the disorder during perimenopause (the transition period before menopause) is lacking [1–3]. Symptoms of perimenopause include somatic symptoms – for example, insomnia, vasomotor and sexual discomfort, and psychological symptoms, such as depression and anxiety [4]. Women who have a history of mental illness, such as mood, anxiety, and stress-related disorders, including post-traumatic stress disorder (PTSD), are at risk of relapse of their mental illness and of a decline in their physical health during this period of life [5–7]. Women with severe and persistent mental illness may be particularly susceptible to perimenopausal symptoms [8]. Similarly, women without any history of mental illness may experience a deterioration in mental and physical health during the perimenopausal period [5, 6, 9]. While most women experience mild-to-moderate adverse symptoms during perimenopause, some encounter severe or very severe symptoms, significantly impacting their health and quality of life [10]. Several socioeconomic and lifestyle-related factors have been found to act as risk factors for severe perimenopausal symptoms – for example, stressful life events and smoking – while being in a relationship and higher education level appear to act as protective factors for adverse symptoms of perimenopause [11].

Given the elevated prevalence of mood-, anxiety-, and stress-related disorders in women with ADHD [12, 13], and also certain socioeconomic and lifestyle-related risk factors [14–16], it is possible that individuals diagnosed with ADHD could be more affected by adverse symptoms of perimenopause [5, 12, 17, 18]. Moreover, emerging evidence from a large-scale genomic analysis indicates that women with ADHD may go through menopause earlier than women without ADHD [19]. Premature menopause (before 40 years) and early menopause (age 40–45 years) are associated with long-term health risks, including cardiovascular disease, neurologic disorders, osteoporosis, psychosexual dysfunction, and mood disorders [20], underscoring the importance of elucidating the interplay between ADHD and the onset of perimenopause.

During perimenopause, women may also experience subjective cognitive difficulties – for example, impaired executive functioning, attention, and memory problems – symptoms similar to the core symptoms of ADHD [5, 17, 21–25]. Reproductive hormones, such as estrogen, affect brain organization and play a crucial role in various cognitive functions, including learning and memory and mood regulation [21, 26]. They may also act as a protective factor against psychiatric symptoms like depression [5, 27]. Research on how reproductive hormones impact the symptoms and treatment of women with ADHD is lacking [26, 28], but emerging evidence has suggested that reproductive hormones may affect ADHD symptoms in women. ADHD symptoms have been found to fluctuate with hormonal changes across the menstrual cycle, suggesting that they may be more variable than previously assumed [29]. Women often complain about increased ADHD symptoms and reduced efficacy of stimulant treatment during the premenstrual period when estrogen levels are low [24, 29]. Moreover, the results of a study conducted by Dorani et al. suggested a heightened prevalence of hormone-related mood symptoms across the lifespan of women with ADHD [17].

Considering the interplay between estrogen and dopamine levels with ADHD symptoms, it is plausible that hormonal fluctuations in perimenopause could exacerbate existing ADHD symptoms or could even lead to a diagnosis of previously unrecognized ADHD [5, 30].

Further research is needed to elucidate the psychological and physical consequences of perimenopause on women with ADHD [14, 28, 31]. In this population-based study, we sought to compare the levels of perimenopausal symptoms among women with and without ADHD across age groups, and to determine whether women with ADHD have a higher prevalence of severe psychological and somatic symptoms of perimenopause, as well as to understand the prevalence of severe ADHD symptoms across the perimenopausal period.

## Methods

### Study population and data source

In this cohort-design study, we used data from the Icelandic Stress-and-Gene-Analysis (SAGA) cohort, a nationwide-representative population-based study, that assessed history of trauma and physical and mental health [10]. In the SAGA cohort, all women aged 18–69 years in 2018 residing in Iceland at the time of the survey were invited to respond to an electronic questionnaire about their demographics and history of trauma and physical and mental health. To participate, women had to understand and be capable of responding in Icelandic and have access to electronic identification. In total, 30,403 women consented to participate in the first wave of the SAGA cohort study in 2018, and of those, 16,750 participated in a follow-up survey in 2024.

In this study, we included all women aged 35–55 years at follow-up (*n* = 5,392) who responded to both baseline and follow-up surveys and specifically completed the Adult ADHD Self-Report Scale (ASRS-v1.1) and the Menopause Rating Scale (MRS) (Supplementary Figure S1). We chose this age range to cover premature perimenopause in the study sample.

For simplicity, we refer to SAGA cohort participants as women, as they had to be registered as women in national registers to enroll and sign an electronic consent.

### Study measures

The history of an ADHD diagnosis was assessed in the first wave and the follow-up survey of the SAGA cohort survey. We ascertained an ADHD diagnosis using self-reported history of an ADHD diagnosis in either the first wave or in the follow-up survey.

We assessed ADHD symptoms using the 6-item screening version of ASRS-v.1.1 measured in the follow-up survey of the SAGA cohort study in 2024 [32–34]. Each item of the ASRS screener is scored on a 5-point response scale of 0 (*never*) to 4 (*very often*). We defined severe ADHD symptoms as scoring ≥18 points on the scale [32–34].

We assessed perimenopausal symptoms over the past month among women in the study population using the MRS, an instrument designed to measure health-related quality of life or severity of complaints in aging women. The MRS consists of 11 items (symptoms) measured in the follow-up survey of the SAGA cohort study in 2024 [35, 36]. Each of the 11 symptoms is measured on a 5-point Likert scale from 0 (*no symptoms*) to 4 (*severe symptoms*) depending on the severity of the symptoms perceived over the last month. MRS has three categories (subdimensions): (1) somatic (hot flushes/sweating, heart discomfort, sleeping problems, and muscle and joint problems); (2) psychological (depressive mood, irritability, anxiety, and tiredness); and (3) urogenital (sexual problems, bladder problems, and vaginal dryness). The composite scores for each of the dimensions are based on adding up the scores of each item of the respective dimensions. The composite score (total score) is the sum of the dimension scores [37]. The total score of the MRS is between 0 (asymptomatic) and 44 (the highest degree of complaints). We defined severe perimenopausal symptoms as a score of ≥17 on the total scale, ≥7 on the psychological subdimension, ≥9 on the somatic subdimension, and ≥4 on the urogenital subdimension [37, 38].

We assessed general physical symptoms using the 15-item somatic symptom module of the Patient Health Questionnaire (PHQ-15), measured in the follow-up survey of the SAGA cohort study in 2024 [39, 40]. Each of the 15 somatic symptoms is scored from 0 (*not bothered at all*) to 2 (*bothered a lot*) over the past 4 weeks. We defined severe general physical symptoms as a score of ≥15 on the PHQ-15, which includes questions on pain, dizziness, heart problems, breathing problems, sexual problems, digestive problems, tiredness, and sleep problems [39, 40].

### Covariates

The SAGA cohort survey included questions on demographic characteristics. Covariates included age, education level, relationship status, smoking status, binge drinking (having four or more drinking units when consuming alcohol), previous psychiatric diagnoses (including a lifetime diagnosis of PTSD), and a question on whether women believed they had entered the perimenopause or not.

### Data analysis

We used R (version 2024.09.0+375) for the statistical analysis. Frequencies and measures of central tendency and dispersion were computed to describe background factors of the population. We described mean scores with standard deviation for ADHD symptoms and determined the prevalence of severe ADHD symptoms using logistic regression models, by age groups and ADHD diagnosis.

We described the mean scores and mean difference with confidence intervals (CIs) for perimenopausal symptoms, including total symptoms, psychological symptoms, somatic symptoms, and urogenital symptoms (MRS) among women with and without ADHD with linear regression models, adjusted for age and with age group as an interaction term. With reference to women of premenopausal and postmenopausal age, we repeated this analysis among women aged 25–30 (*n* = 839) and 60–65 (*n* = 1,487) years.

We contrasted the prevalence of severe perimenopausal symptoms, including severe psychological, somatic and urogenital symptoms (MRS), and of severe general physical symptoms (PHQ-15), among women with and without ADHD, and calculated prevalence ratios (PRs) with 95% CI using modified Poisson regression models [41]. We also fitted logistic regression models to the data to inspect modeling assumptions and goodness of fit using the Hosmer–Lemeshow test. We adjusted these models for age because of the different mean age of women with and without ADHD. We then adjusted the models for other confounding variables, that is, for education level, relationship status, smoking, and binge drinking. We removed 260 individuals (5%) with unknown/missing responses in this analysis (Table 1) and decided not to use multiple imputation, as previous studies using data from the SAGA cohort study have demonstrated that it does not lead to any significant changes in results [10, 42]. We used the “emmeans” package in R for these analyses [43].

**Table 1.** Characteristics of women with and without ADHD in the SAGA cohort study, number (%), or mean ± standard deviation (SD)

	ADHD (*N* = 535)	Non-ADHD (*N* = 4,857)
Age		
Mean (SD)	43.6 (5.62)	45.7 (5.87)
Median (min, max)	43.0 (35.0, 55.0)	46.0 (35.0, 55.0)
Age group (years)		
35–39	153 (28.6%)	923 (19.0%)
40–44	156 (29.2%)	1,145 (23.6%)
45–49	130 (24.3%)	1,226 (25.2%)
50–55	96 (17.9%)	1,563 (32.2%)
Highest education		
Primary	66 (12.3%)	304 (6.3%)
Secondary	110 (20.6%)	665 (13.7%)
Vocational	156 (29.2%)	1,623 (33.4%)
University	165 (30.8%)	2,102 (43.3%)
Unknown/missing	38 (7.1%)	163 (3.4%)
Relationship status		
Married or in a relationship	419 (78.3%)	4,212 (86.7%)
Single	114 (21.3%)	630 (13.0%)
Unknown/missing	2 (0.4%)	15 (0.3%)
Smoking		
Current smoker	89 (16.6%)	358 (7.3%)
Previous smoker	273 (51.0%)	1,721 (35.4%)
Never	170 (31.8%)	2,747 (56.6%)
Unknown/missing	3 (0.5%)	31 (0.6%)
Binge drinking		
Never	190 (35.5%)	1,689 (34.8%)
Monthly or less	277 (51.8%)	2,773 (57.1%)
Weekly or more	65 (12.2%)	380 (7.8%)
Unknown/missing	3 (0.6%)	15 (0.3%)
Other diagnosis		
Lifetime PTSD	225 (42.1%)	501 (10.3%)
ADHD symptom score		
ASRS mean score (SD)	14.5 (4.9)	8.8 (5.1)
Reported perimenopause		
Self-reported perimenopause	252 (47.1%)	2,669 (55.0%)

Abbreviations: ADHD, attention-deficit/hyperactivity disorder; ASRS, Adult ADHD Self-Report Scale; GAD, generalized anxiety disorder; PTSD, post-traumatic stress disorder; SD, standard deviation.

#### Secondary analyses

First, considering that a high proportion of the women in our sample had a lifetime diagnosis of PTSD (the SAGA cohort is a study on trauma history), as a secondary analysis, we stratified the main analysis by lifetime PTSD diagnosis. Second, we repeated the main analysis, including only women who self-reported that they had entered the perimenopause. Third, to rule out potential misclassification of reported ADHD diagnoses, we repeated the main analyses using severe current ADHD symptoms as the exposure variable instead of an ADHD diagnosis.

The SAGA cohort study was approved by the National Bioethics Committee of Iceland (no. 17-238) and the Icelandic Data Protection Authority.

### Role of the funding source

The funders of the study had no role in study design, data collection, data analysis, data interpretation, writing of the manuscript, or the decision to submit.

### Results

Among 5,392 women aged 35–55 years, we identified 535 (9.9%) with ADHD and 4,857 (90.1%) without ADHD. Women with ADHD were on average 2 years younger than women without ADHD (43.6 vs. 45.7 years; Table 1). Compared with women without ADHD, women with ADHD were more likely to have a lower educational level (12.3% vs. 6.3% with primary education as highest education level and 20.6% vs. 13.7% with secondary education as highest education level). They were more likely to be single (21.3% vs. 13.0%), to smoke (16.6% vs. 7.3%), and to binge drink weekly or more often (12.2% vs. 7.8%). Women with ADHD were more likely to have a lifetime PTSD diagnosis (42.1% vs. 10.3%). The average score on ASRS was 14.5 points for women with ADHD and 8.8 points for women without ADHD. Among women with ADHD, 47.1% of the women reported having entered the perimenopause compared to 55.0% of women without ADHD (Table 1).

**Figure 1.**
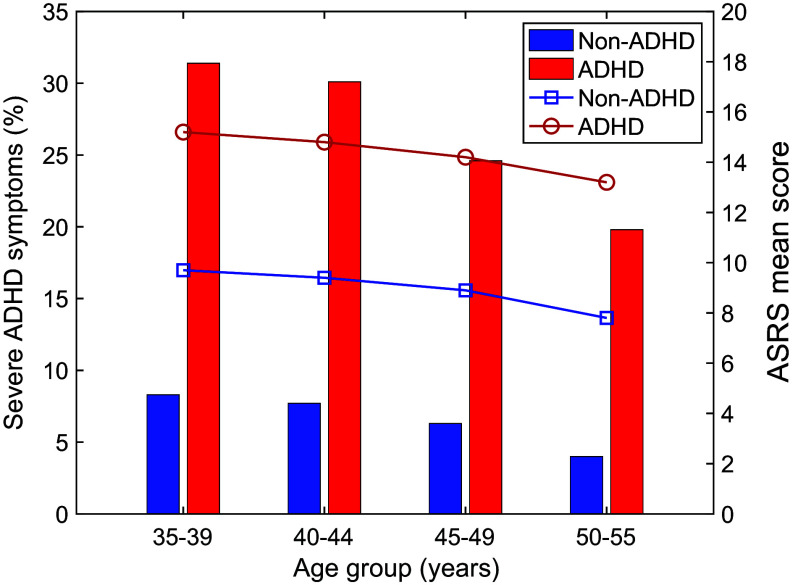
Proportion of women with severe current ADHD symptoms, by age group, among women with and without ADHD (columns), and mean ADHD symptom score on ASRS, across age groups, among women with and without ADHD (lines). Abbreviations: ADHD, attention deficit/hyperactivity disorder; ASRS, Adult ADHD Self-Report Scale.

In our population, 8.3% of women reported severe current ADHD symptoms. The proportion of women reporting severe current ADHD symptoms was highest in the youngest age group, 35–39 years (11.6%), and decreased with age, both among women with and without an ADHD diagnosis (Figure 1).

Women with ADHD had a significantly higher total perimenopausal symptom score (MRS) than women without ADHD (mean score 18.0 vs. 13.0, *p* < 0.01) and across all age groups (Table 2). The difference was most pronounced among women aged 35–39 years, both overall (mean total score difference 6.5 points, *p* < 0.01) and by MRS subdimensions: psychological symptoms (mean score difference 2.8 points, *p* < 0.01), somatic symptoms (mean score difference 2.5 points, *p* < 0.01), and urogenital symptoms (mean score difference 1.2 points, *p* < 0.01). Perimenopausal symptom scores were highest at age 35–39 years among women with ADHD but at age 45–49 years among women without ADHD (Table 2).

**Table 2.** Mean scores and mean score differences with 95% confidence intervals on perimenopausal symptoms measured by the MRS and MRS subdimensions, by ADHD diagnosis, adjusted for age, and by women’s age group

Total for age 35–55 years (age-adjusted)		ADHD	Non-ADHD	Mean score difference (CI)
(*n* = 5,392)	**Total score**	**18.0 (17.4–18.6)**	**13.0 (12.8–13.2)**	5.0 (4.3–5.7)**
	Psychological	8.1 (7.8–8.5)	5.7 (5.6–5.8)	2.4 (2.1–2.8)**
	Somatic	6.7 (6.4–6.9)	4.9 (4.8–5.0)	1.8 (1.5–2.0)**
	Urogenital	3.2 (3.0–3.4)	2.4 (2.3–2.5)	0.8 (0.6–1.1)**
**Age groups**				
25–30 years (*n* = 839)	**Total score**	**18.0 (17.0–19.1)**	**12.4 (11.8–13.0)**	5.6 (4.4–6.8)**
	Psychological	8.9 (8.4–9.4)	6.6 (6.3–6.9)	2.3 (1.7–2.9)**
	Somatic	6.1 (5.6–6.5)	3.9 (3.7–4.2)	2.2 (1.6–2.7)**
	Urogenital	3.1 (2.8–3.4)	1.9 (1.7–2.1)	1.2 (0.9–1.6)**
35–39 years (*n* = 1,076)	**Total score**	**19.0 (17.8–20.2)**	**12.5 (12.1–13.0)**	6.5 (5.2–7.8)**
	Psychological	9.1 (8.5–9.8)	6.3 (6.0–6.6)	2.8 (2.2–3.5)**
	Somatic	6.7 (6.2–7.2)	4.2 (4.0–4.4)	2.5 (1.9–3.0)**
	Urogenital	3.2 (2.8–3.6)	2.0 (1.9–2.2)	1.2 (0.8–1.5)**
40–44 years (*n* = 1,301)	**Total score**	**18.4 (17.2–19.6)**	**13.1 (12.7–13.5)**	5.3 (4.0–6.6)**
	Psychological	8.8 (8.2–9.4)	6.2 (5.9–6.4)	2.6 (2.0–3.3)**
	Somatic	6.5 (6.0–7.0)	4.7 (4.6–4.9)	1.8 (1.3–2.3)**
	Urogenital	3.1 (2.8–3.5)	2.2 (2.1–2.3)	0.9 (0.5–1.3)**
45–49 years (*n* = 1,356)	**Total score**	**16.7 (15.3–18.0)**	**13.4 (12.9–13.8)**	3.3 (1.9–4.7)**
	Psychological	7.2 (6.6–7.9)	5.7 (5.5–5.9)	1.5 (0.8–2.2)**
	Somatic	6.5 (6.0–7.1)	5.2 (5.0–5.4)	1.3 (0.8–1.9)**
	Urogenital	2.9 (2.5–3.4)	2.5 (2.3–2.6)	0.5 (0.0–0.9)*
50–55 years (*n* = 1,659)	**Total score**	**17.6 (16.1–19.1)**	**12.9 (12.5–13.2)**	4.7 (3.2–6.3)**
	Psychological	7.4 (6.6–8.1)	4.7 (4.5–4.9)	2.7 (1.9–3.5)**
	Somatic	6.7 (6.1–7.3)	5.4 (5.2–5.6)	1.3 (0.6–1.9)**
	Urogenital	3.6 (3.0–4.1)	2.8 (2.7–2.9)	0.8 (0.2–1.3)**
60–65 years (*n* = 1,487)	**Total score**	**17.2 (15.5–18.8)**	**11.1 (10.8–11.5)**	6.1 (4.4–7.7)**
	Psychological	6.8 (6.0–7.5)	3.5 (3.3–3.6)	3.3 (2.5–4.1)**
	Somatic	6.3 (5.7–7.0)	4.9 (4.8–5.1)	1.4 (0.7–2.1)**
	Urogenital	4.1 (3.5–4.7)	2.8 (2.6–2.9)	1.3 (0.7–2.0)**

Abbreviations: ADHD, attention deficit/hyperactivity disorder; CI, confidence interval; MRS, Menopause Rating Scale.

*Note*: Test of effect modification by age *p <* 0.05.

* *p* < 0.05.

** *p* < 0.01.

**Figure 2.**
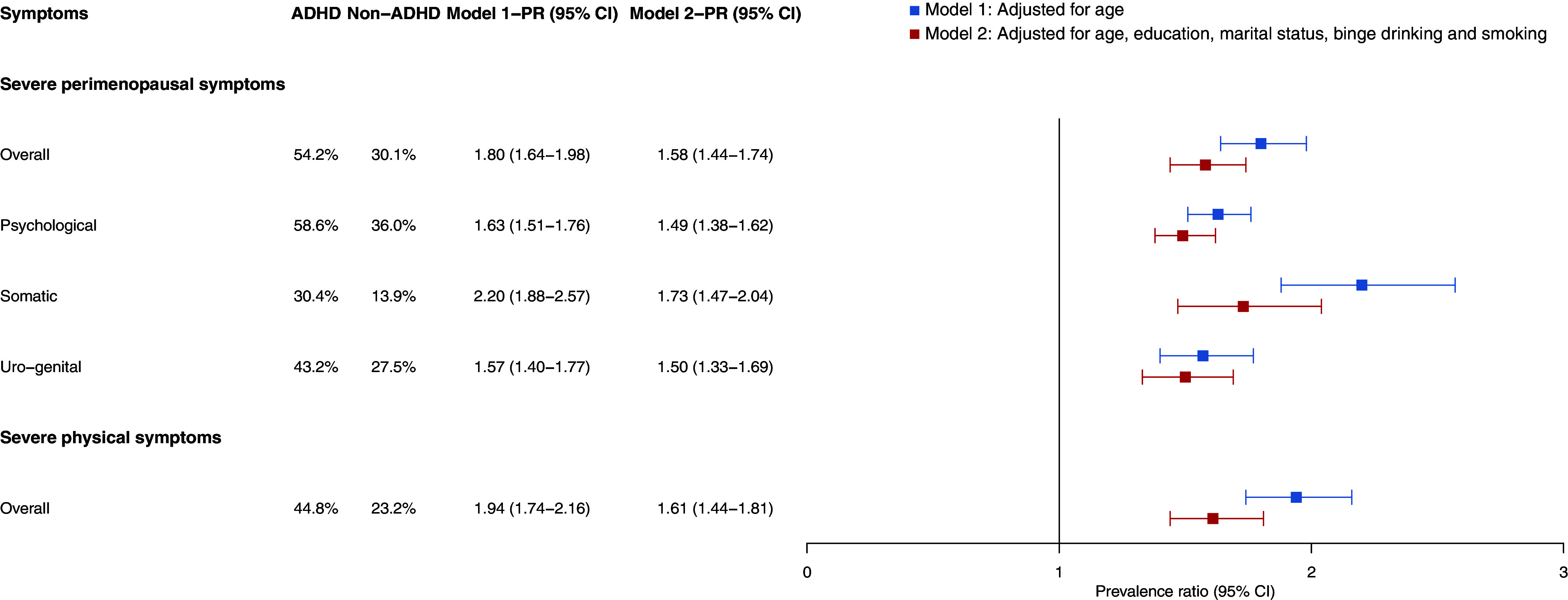
Age-adjusted prevalence and prevalence ratios (PRs) of severe perimenopausal symptoms by subdimension (MRS) and severe general physical symptoms (PHQ-15) among women of perimenopausal age according to ADHD diagnosis. Complete case analysis. Abbreviations: ADHD, attention deficit/hyperactivity disorder; PR, prevalence ratio, CI, confidence interval.

The models were considered to have a good fit to the data (*p* > 0.05, Hosmer–Lemeshow test). The age-adjusted prevalence of severe perimenopausal symptoms was higher among women with ADHD than among those without ADHD (Figure 2), both overall (54.2% vs. 30.1%; PR = 1.80, 95% CI = 1.64–1.98) and by MRS subdimensions, severe psychological symptoms (58.6% vs. 36.0%; PR = 1.63, 95% CI = 1.51–1.76), severe somatic symptoms (30.4% vs. 13.9%; PR = 2.20, 95% CI = 1.88–2.57), and severe urogenital symptoms (43.2% vs. 27.5%; PR = 1.57, 95% CI = 1.40–1.77). Similarly, women with ADHD had a higher age-adjusted prevalence of severe general physical symptoms (PHQ-15) than those without ADHD (44.8% vs. 23.2%; PR = 1.94, 95% CI = 1.74–2.16). All PRs remained elevated, but slightly attenuated, after adjusting for education level, relationship status, binge drinking, and smoking.

#### Secondary results

Stratifying the analysis by lifetime PTSD diagnosis, we observed that among women without a lifetime PTSD diagnosis, the prevalence ratios for severe perimenopausal symptoms, both overall and on all subdimensions attenuated, but remained significant, even after adjusting for education level, relationship status, binge drinking, and smoking (Supplementary Figure S2). Among women with a lifetime PTSD diagnosis, we found that the prevalence of severe perimenopausal symptoms, both psychological and somatic, was elevated both among women with and without ADHD. However, women with ADHD still had significantly higher prevalences of severe perimenopausal symptoms compared to women without ADHD (Supplementary Figure S3).

The prevalence ratios for severe perimenopausal symptoms attenuated slightly but remained significant when the analysis was repeated among women who self-reported that they had entered the perimenopause. This was mainly due to slightly higher prevalences of severe perimenopausal symptoms among women without ADHD in this subgroup of women (Supplementary Figure S4).

Repeating the main analyses with severe current ADHD symptoms as the exposure variable, we found that women with severe ADHD symptoms (*n* = 450, 8.3%) had higher scores on the MRS (Supplementary Table S1) and higher prevalence of severe perimenopausal symptoms, both overall (72.8% vs. 28.9%, PR = 2.53, 95% CI = 2.35–2.73) and on all subdimensions, compared with women without severe ADHD symptoms. The association was even stronger than we observed in the main analysis among women with an ADHD diagnosis (Supplementary Figure S5).

## Discussion

This population-based study is, to our knowledge, the first to assess differences in perimenopausal symptoms among women with and without ADHD. Our findings suggest a considerably higher symptom burden, including impairing psychological and somatic symptoms, among women with ADHD, compared to those without ADHD. These differences were most pronounced at age 35–39 years, suggesting an onset of perimenopause up to 10 years earlier in women with ADHD than in the average. Moreover, we observed severe current ADHD symptoms in a large proportion of women of perimenopausal age, both among those with and without an ADHD diagnosis.

In our data, women with ADHD scored higher on all measured subdimensions of perimenopausal symptoms measured on the MRS, psychological symptoms including depressive symptoms, irritability, fatigue, and anxiety; urogenital symptoms such as sexual discomfort, vaginal dryness, and bladder problems; and somatic symptoms like hot flushes, heart discomfort (unusual awareness of heartbeat, extra beats, rapid heartbeat, and palpitations), sleep problems, joint aches, and other general physical symptoms measured by the PHQ-15, including headaches and digestive problems. Previous research has established associations between ADHD in women and psychiatric comorbidities like anxiety and mood disorders [3, 12, 17, 18, 44, 45]. Associations between ADHD and adverse physical health have been found, but fewer studies have examined this association [12, 18, 46–48]. Interestingly, according to our results, the association between ADHD and physical symptoms seemed to be particularly pronounced among women of perimenopausal age. It is noteworthy that women with ADHD of premenopausal and postmenopausal age, that is, aged 25–30 and 60–65 years, reported high symptom scores on the MRS, in accordance with what we observed among women of perimenopausal age. This observation suggests that the MRS scale may not exclusively be measuring perimenopausal symptoms, but also general psychological and physical symptoms arising in women with ADHD across age groups.

Of note is our finding that women with ADHD reported the highest perimenopausal symptom levels at age 35–39 years, while these were highest at age 45–49 years among women without ADHD. This is in line with the results of the first genome-wide meta-analysis study by Demontis et al., indicating that women with ADHD have an earlier age of menopause than women without ADHD [19]. Further research is needed to understand the impact of hormonal replacement therapy and/or stimulant treatment on the connection between ADHD and perimenopausal symptoms. Menopausal hormonal replacement therapy has been shown to reduce the risk of adverse health outcomes of early menopause [49].

Our results showed that among women diagnosed with ADHD, a third reported severe current ADHD symptoms at ages 35–39 (31.4%) and 40–44 (30.1%) years. This suggests a high prevalence of impairing ADHD symptoms coinciding with the critical life phase of women before entering menopause, as indicated by the ongoing work of Wasserstein et al. [50]. While severe ADHD symptoms were lower among older women, the prevalence was still 25 and 20%, respectively, at ages 45–49 and 50–55 years among women with ADHD in our sample. This emphasizes the importance of considering ADHD as a lifelong disorder in women. Among women aged 35–44 years without an ADHD diagnosis, the proportion reporting severe ADHD symptoms was around 8% and lowered with increased age. We were unable to determine whether the women who had not been diagnosed with ADHD, but reported severe ADHD symptoms, had impairing ADHD symptoms in childhood, whether the symptoms emerged in later years, or what caused severe ADHD symptoms in these age groups. However, it seems plausible that fluctuating reproductive hormones played a significant role in this association by exacerbating ADHD symptoms [50].

In our data, women with ADHD were more likely to have been diagnosed with PTSD at some point in their lives than women without ADHD. To rule out the potential role of PTSD on our results and because of shared symptomology in PTSD and perimenopause [51], we conducted an analysis stratified by lifetime PTSD diagnosis, which revealed that the association between ADHD and severe perimenopausal symptoms was slightly attenuated but remained significant among women without a lifetime PTSD diagnosis.

Strengths of this study include a relatively large sample size that is representative of the sociodemographics of the female adult population in Iceland regarding demographic characteristics [10]. Validated instruments were used to measure ADHD and perimenopausal symptoms, and the stratification by lifetime PTSD diagnosis provided valuable insight into comorbidity. However, there are several limitations to be noted. First, given the cross-sectional nature of measurements, we could not assert a causal relationship between ADHD and more severe perimenopausal symptoms. However, since ADHD develops in childhood [52], we could assert that the symptoms of the disorder precede perimenopausal symptoms in time. Second, some symptoms measured in the MRS are not exclusive to the perimenopause – for example, depression, anxiety, and irritability. We administered the scale to all women irrespective of age or whether they believed they had entered the perimenopause or not. Therefore, we cannot be certain that we are exclusively measuring perimenopausal symptoms. However, we addressed this by examining perimenopausal symptoms in younger and older age groups and by stratifying the analyses by women who reported that they were perimenopausal. When restricting the analysis to women who self-reported being perimenopausal, the prevalence ratios attenuated slightly; however, the associations remained robust, with women with ADHD continuing to show higher prevalences of severe perimenopausal symptoms, both overall and across all subdimensions. In the current study, we did not have data on previous or current pharmacological treatment. In future studies, it would be interesting to examine the impact of medications, such as psychostimulants and other psychotropics, menopausal hormonal replacement therapy, as well as oral contraceptives, on perimenopausal symptoms among women with ADHD. Third, diagnoses of ADHD were self-reported, which could lead to misclassification of the reported diagnoses; it is difficult to determine the full implications of this in our findings, given that diagnoses may be both under- and overreported. In our sample, 9.9% of the women reported a diagnosis of ADHD, which is higher than the reported prevalence of ADHD among female adults in the general population [2]. We addressed the potential misclassification of ADHD in a sensitivity analysis examining women with severe ADHD symptoms (independent of having a diagnosis). The results of this analysis suggested an even stronger association between having severe ADHD symptoms and severe perimenopausal symptoms than observed in the main analysis. Moreover, women identified as having an ADHD diagnosis in our sample had, on average, higher scores on the ASRS scale than those without ADHD, suggesting a correlation between reported diagnosis and symptoms of ADHD. Fourth, as the SAGA cohort study is a study on trauma history, it could be hypothesized that women who had experienced trauma were more likely to participate in the study than women who had not. However, the SAGA cohort is representative of the Icelandic female population in terms of sociodemographic factors – for example, age, education, income, and region of residence [10].

In conclusion, our findings shed light on the enduring burden of ADHD on women, especially during the perimenopause. Women with ADHD are at risk of developing severe psychological and physical comorbidities of the perimenopause and may start to experience perimenopausal symptoms at an earlier age than women without ADHD. Moreover, women with ADHD have a high prevalence of severe ADHD symptoms during the perimenopausal period. These findings underscore the importance of developing guidelines for the treatment and care of perimenopausal women with ADHD, including tailored interventions to address their unique needs.

## Supporting information

10.1192/j.eurpsy.2025.10101.sm001 Jakobsdóttir Smári et al. supplementary material Jakobsdóttir Smári et al. supplementary material

## Data Availability

The data used in this study were compiled in the Stress-And-Gene-Analysis (SAGA) cohort. These data cannot be made publicly available, according to Icelandic data protection laws and the terms of approval for the current study that were stipulated by the National Bioethics Committee of Iceland. The SAGA cohort contains extremely sensitive data, and all use of data is subject to NBC approval (email: vsn@vsn.is). Interested researchers can obtain access to de-identified data by submitting a proposal to the SAGA cohort data management board (email: afallasaga@hi.is), which will assist with submitting a request to the NBC to obtain data from the study.

## Supplementary material

The supplementary material for this article can be found at http://doi.org/10.1192/j.eurpsy.2025.10101.
