# Supplementary material for: Perimenopausal symptoms in women with and without ADHD: A population-based cohort study
Source: Eur Psychiatry. 2025 Sep 4;68(1):e133. doi: 10.1192/j.eurpsy.2025.10101 (PMC12538516; doi:10.1192/j.eurpsy.2025.10101)
Supplement: Jakobsdóttir Smári et al. supplementary material [file S0924933825101016sup001.zip › Supplementary tables.docx]

**Supplemental table 1.** Mean scores and mean score difference with 95% confidence intervals on perimenopausal symptoms measured by the MRS and MRS subdimensions, by a high score on the ASRS, with severe symptoms defined as a score ≥18, adjusted for age.

| **Total** |  | **Severe ADHD symptoms** | **Non-severe ADHD symptoms** | **Mean score difference (CI)** |
| --- | --- | --- | --- | --- |
| (n=5392) | **Total score** | **21.4 (20.7-22.1)** | **12.8 (12.5-13.0)** | **8.7 (7.9-9.4) **** |
|  | Psychological | 9.7 (9.4-10.1) | 5.6 (5.5-5.7) | 4.1 (3.8-4.5) ** |
|  | Somatic | 7.8 (7.5-8.1) | 4.8 (4.7-4.9) | 3.0 (2.7-3.3) ** |
|  | Uro-genital | 3.9 (3.6-4.1) | 2.3 (2.3-2.4) | 1.5 (1.3-1.8) ** |

**p* < 0.05, ***p* < 0.01

**Abbreviations**: *ADHD* Attention Deficit/Hyperactivity Disorder, *CI* confidence interval, *MRS* Menopause Rating Scale
