# Supplementary material for: Perimenopausal symptoms in women with and without ADHD: A population-based cohort study
Source: Eur Psychiatry. 2025 Sep 4;68(1):e133. doi: 10.1192/j.eurpsy.2025.10101 (PMC12538516; doi:10.1192/j.eurpsy.2025.10101)
Supplement: Jakobsdóttir Smári et al. supplementary material [file S0924933825101016sup001.zip › Supplemental figure 1.pdf]

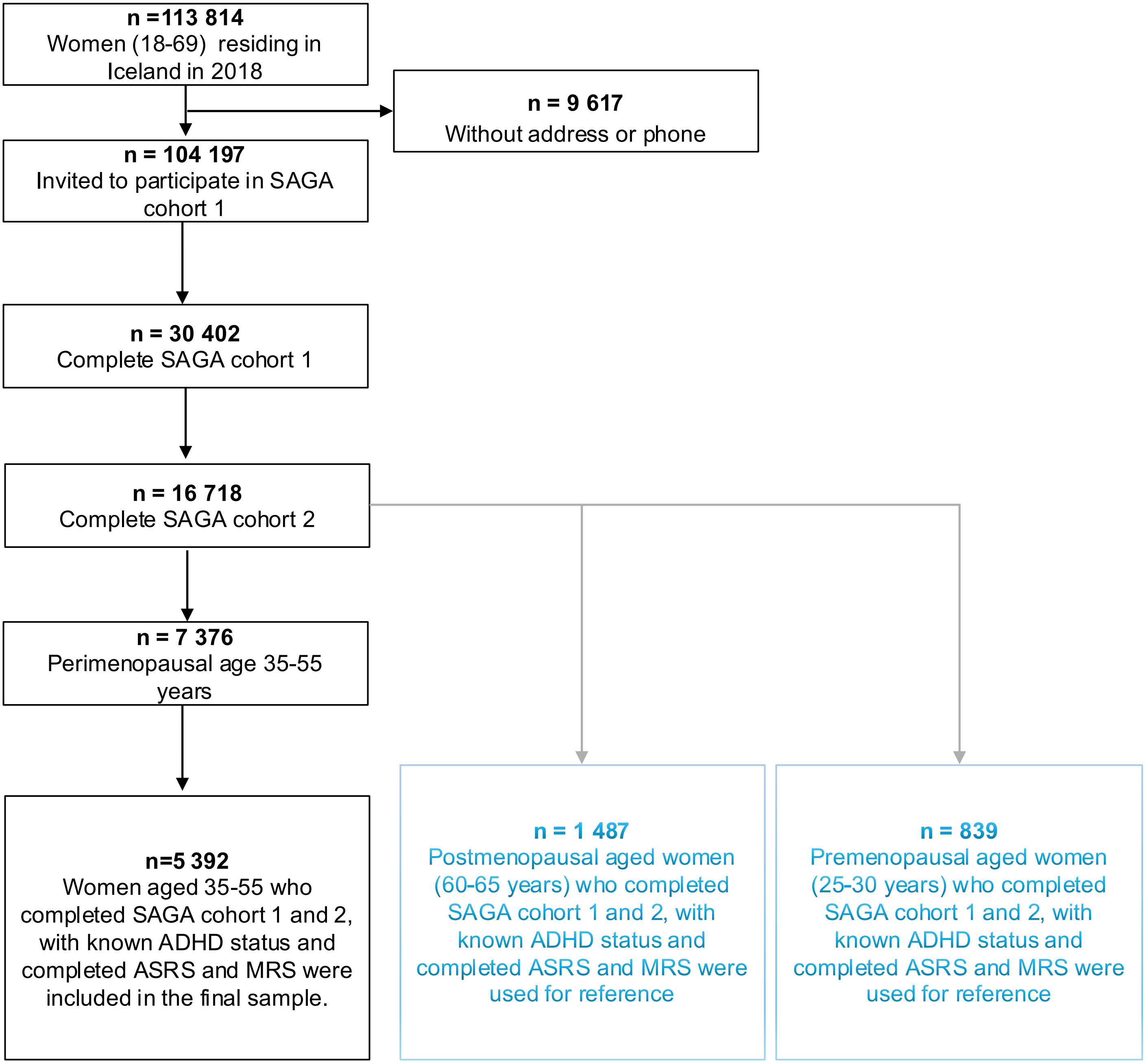

n =113 814
Women (18-69) residing in Iceland in 2018
n = 9 617
Without address or phone
n = 104 197
Invited to participate in SAGA cohort 1
n = 30 402
Complete SAGA cohort 1
n = 16 718
Complete SAGA cohort 2
n = 7 376
Perimenopausal age 35-55 years
n=5 392
Women aged 35-55 who completed SAGA cohort 1 and 2, with known ADHD status and completed ASRS and MRS were included in the final sample.
n = 1 487
Postmenopausal aged women (60-65 years) who completed SAGA cohort 1 and 2, with known ADHD status and completed ASRS and MRS were used for reference
n = 839
Premenopausal aged women (25-30 years) who completed SAGA cohort 1 and 2, with known ADHD status and completed ASRS and MRS were used for reference
