# Supplementary material for: Perimenopausal symptoms in women with and without ADHD: A population-based cohort study
Source: Eur Psychiatry. 2025 Sep 4;68(1):e133. doi: 10.1192/j.eurpsy.2025.10101 (PMC12538516; doi:10.1192/j.eurpsy.2025.10101)

Symptoms	ADHD	Non–ADHD	Model 1–PR (95% CI)	Model 2–PR (95% CI)
**Severe perimenopausal symptoms**				
Overall	41.9%	27.7%	1.51 (1.29–1.78)	1.38 (1.16–1.63)
Psychological	51.6%	33.9%	1.52 (1.34–1.72)	1.43 (1.26–1.62)
Somatic	22.4%	12.1%	1.85 (1.42–2.40)	1.56 (1.18–2.05)
Uro–genital	34.8%	26.2%	1.33 (1.10–1.61)	1.29 (1.06–1.57)
**Severe physical symptoms**				
Overall	36.0%	21.1%	1.71 (1.42–2.04)	1.45 (1.21–1.75)

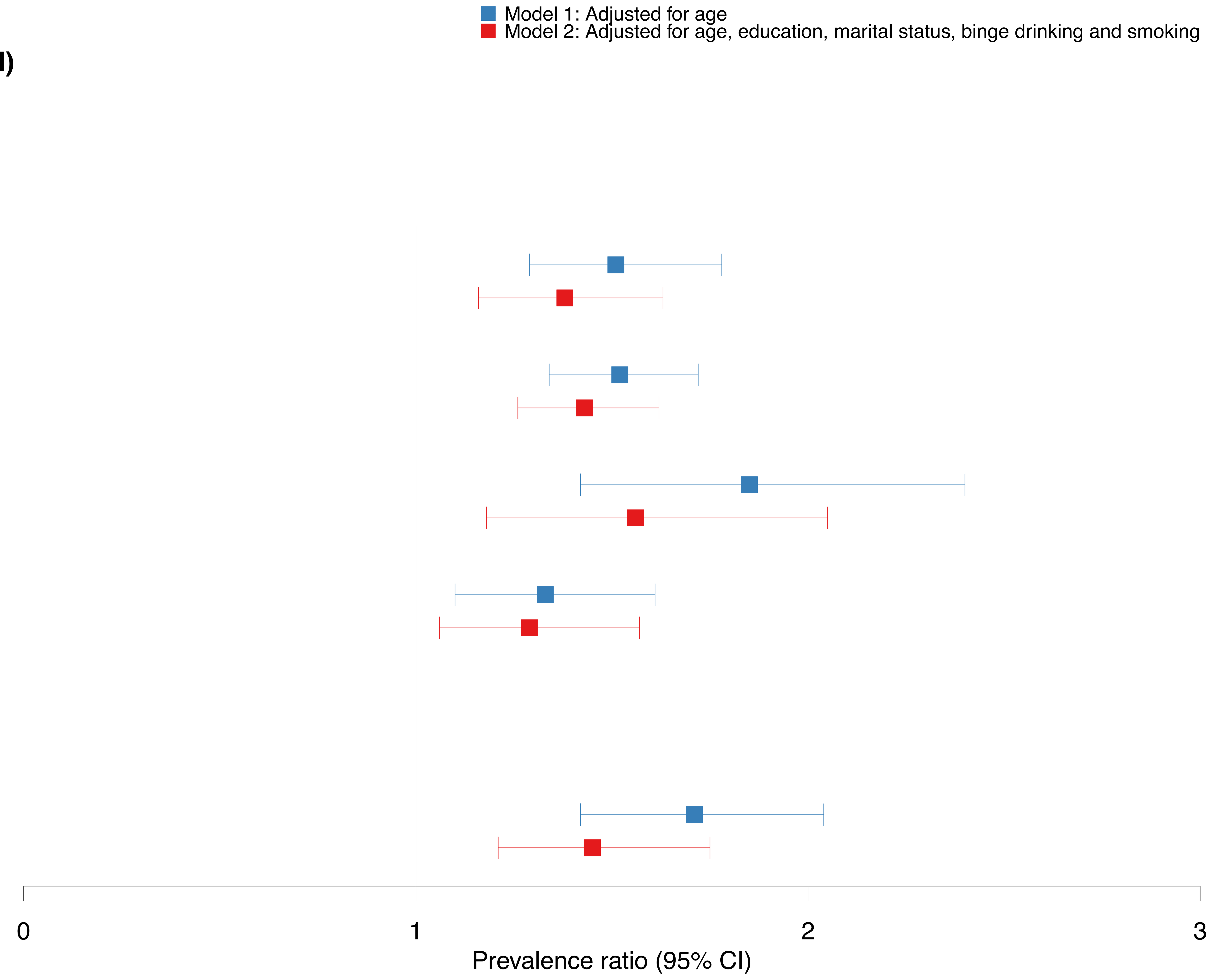

Supplement: Jakobsdóttir Smári et al. supplementary material [file S0924933825101016sup001.zip › Supplemental figure 2.pdf]
