# Supplementary material for: Perimenopausal symptoms in women with and without ADHD: A population-based cohort study
Source: Eur Psychiatry. 2025 Sep 4;68(1):e133. doi: 10.1192/j.eurpsy.2025.10101 (PMC12538516; doi:10.1192/j.eurpsy.2025.10101)

Symptoms	ADHD	Non–ADHD	Model 1–PR (95% CI)	Model 2–PR (95% CI)
**Severe perimenopausal symptoms**				
Overall	67.9%	52.1%	1.31 (1.14–1.49)	1.20 (1.05–1.38)
Psychological	67.6%	54.3%	1.24 (1.10–1.41)	1.19 (1.04–1.36)
Somatic	42.9%	29.1%	1.48 (1.18–1.84)	1.24 (0.99–1.55)
Uro–genital	53.2%	39.0%	1.36 (1.14–1.63)	1.32 (1.09–1.59)
**Severe physical symptoms**				
Overall	59.5%	41.8%	1.43 (1.22–1.67)	1.36 (1.15–1.60)

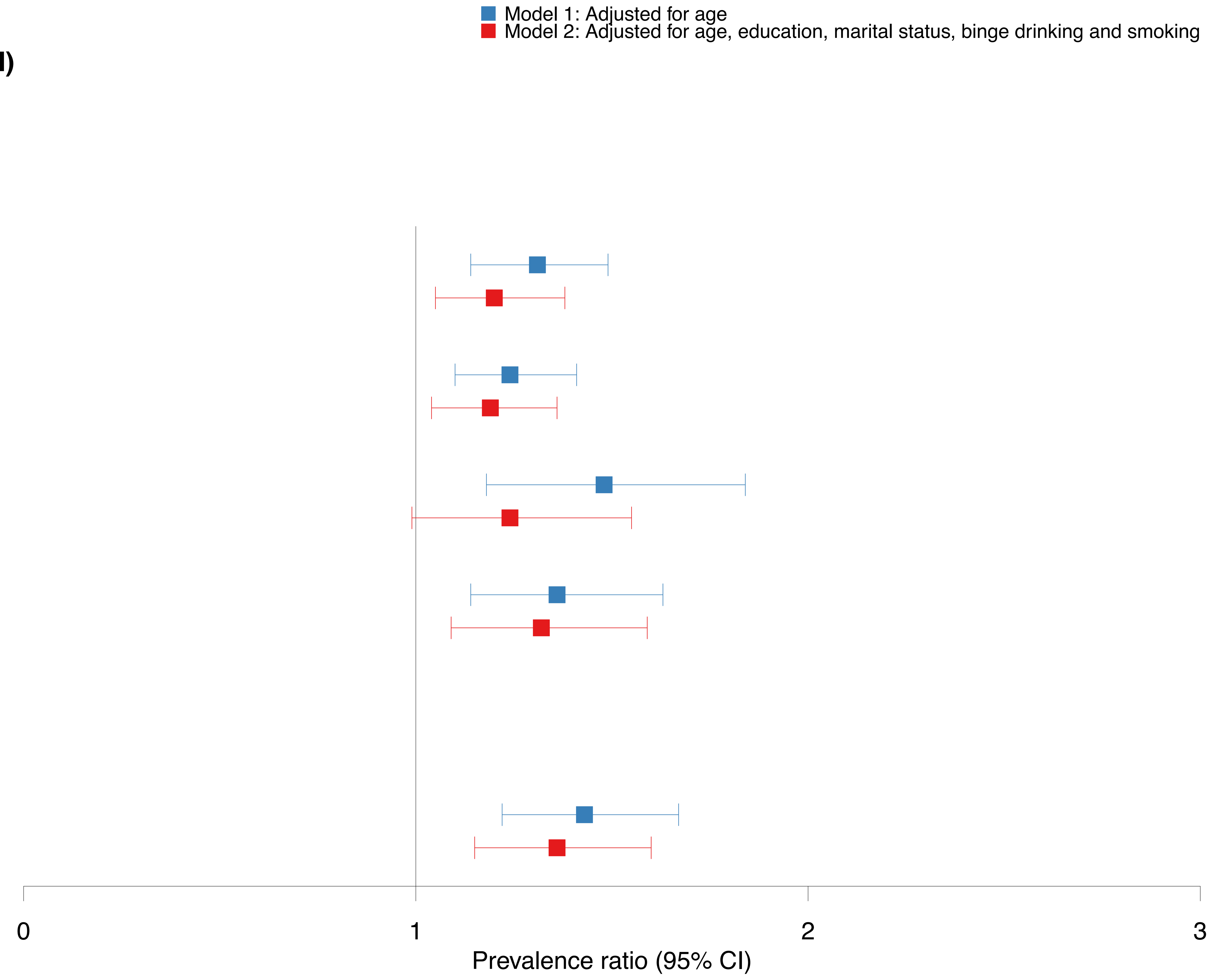

Supplement: Jakobsdóttir Smári et al. supplementary material [file S0924933825101016sup001.zip › Supplemental figure 3.pdf]
