# Supplementary material for: Perimenopausal symptoms in women with and without ADHD: A population-based cohort study
Source: Eur Psychiatry. 2025 Sep 4;68(1):e133. doi: 10.1192/j.eurpsy.2025.10101 (PMC12538516; doi:10.1192/j.eurpsy.2025.10101)

Symptoms	ADHD	Non–ADHD	Model 1 – PR (95% CI)	Model 2 – PR (95% CI)
**Severe perimenopausal symptoms**				
Overall	51.2%	34.1%	1.50 (1.32–1.73)	1.35 (1.16–1.55)
Psychological	54.6%	36.5%	1.49 (1.33–1.68)	1.38 (1.22–1.57)
Somatic	35.7%	17.7%	2.02 (1.64–2.49)	1.59 (1.28–1.99)
Uro–genital	46.4%	31.9%	1.46 (1.23–1.72)	1.37 (1.16–1.63)
**Severe physical symptoms**				
Overall	42.0%	23.7%	1.77 (1.50–2.10)	1.47 (1.23–1.75)

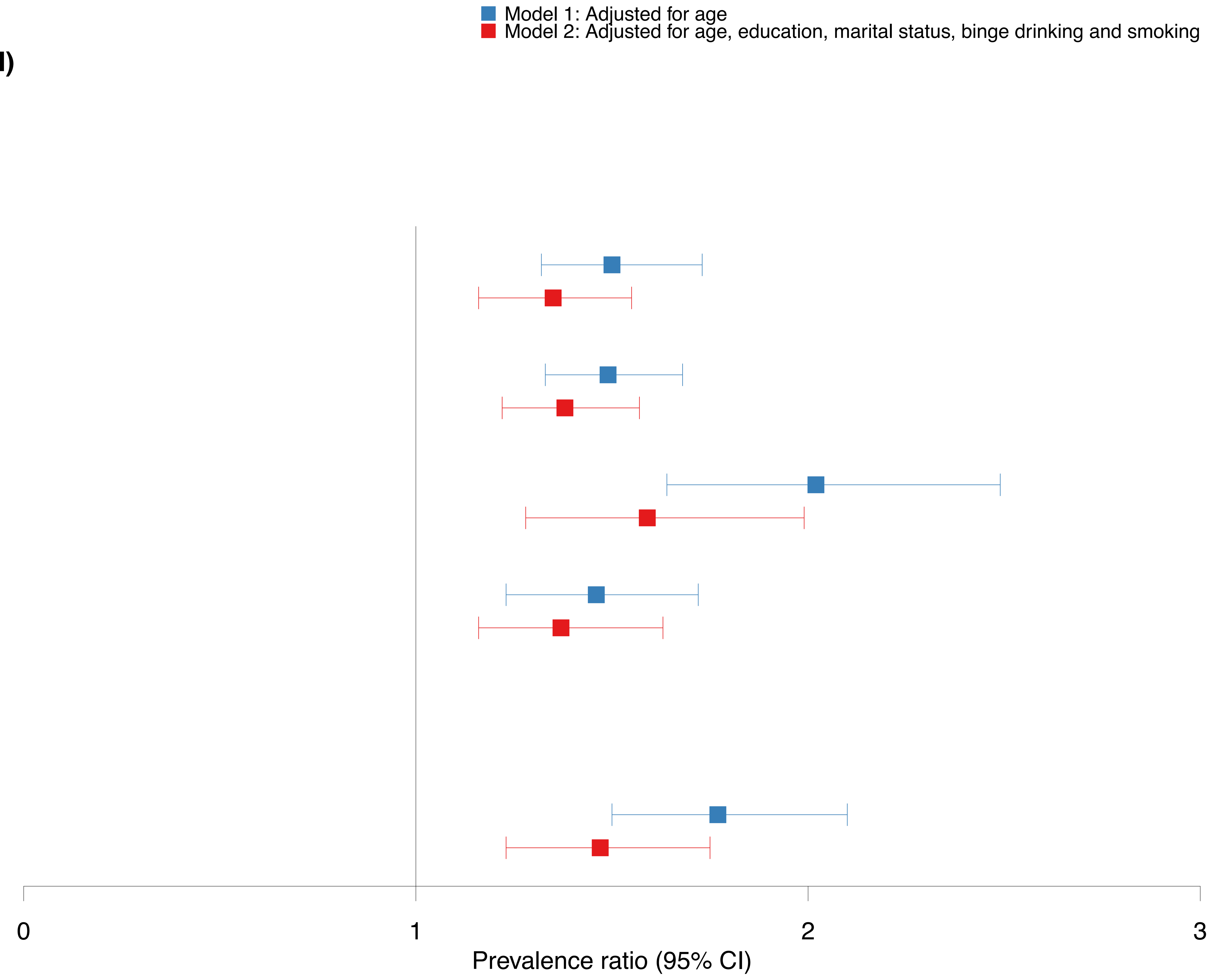

Supplement: Jakobsdóttir Smári et al. supplementary material [file S0924933825101016sup001.zip › Supplemental figure 4.pdf]
