# Supplementary figures and images for: Perimenopausal symptoms in women with and without ADHD: A population-based cohort study
Source: Eur Psychiatry. 2025 Sep 4;68(1):e133. doi: 10.1192/j.eurpsy.2025.10101 (PMC12538516; doi:10.1192/j.eurpsy.2025.10101)

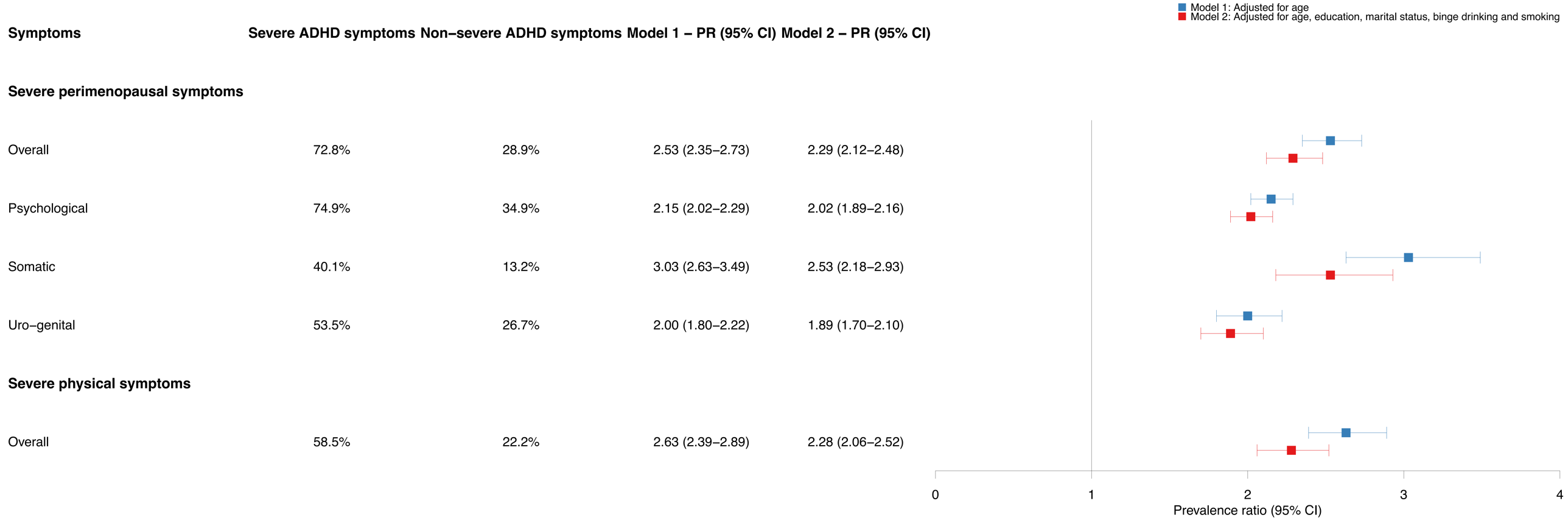

Supplement: Jakobsdóttir Smári et al. supplementary material [file S0924933825101016sup001.zip › Supplemental figure 5.pdf]
